# Surgical Outcomes Following Robotic Single-Site Versus Multiport Hysterectomy for Treatment of Endometrial Cancer: A Systematic Review and Meta-Analysis

**DOI:** 10.7759/cureus.34702

**Published:** 2023-02-06

**Authors:** Emma Schnittka, Nick W Lanpher, Jessica Cushing-murray, Trevor Decker, Praful G Patel

**Affiliations:** 1 Medicine, Alabama College of Osteopathic Medicine, Dothan, USA; 2 Obstetrics and Gynecology, Southeast Health Medical Center, Dothan, USA; 3 Obstetrics and Gynecology, Alabama College of Osteopathic Medicine, Dothan, USA

**Keywords:** endometrial cancers, minimally invasive gynecologic surgery, robotic assited surgery, robotic hysterectomy, single-incision surgery, systematic review and meta analysis

## Abstract

Robotic single-site hysterectomy (RSSH) has emerged as a novel surgical approach for the treatment of endometrial cancer and atypical endometrial hyperplasia (AEH). Current research regarding the benefits of RSSH compared to robotic multiport hysterectomy (RMPH) for these indications has been inconclusive. Our team sought to compare surgical outcomes between these two approaches of robotic hysterectomy via systematic review and meta-analysis to ensure optimal surgical practices. The Preferred Reporting Items for Systematic Reviews and Meta-Analyses (PRISMA) 2020 Checklist guided our review. MEDLINE, Clinicaltrials.gov, and Cochrane Library were searched, yielding 59 results. Articles were filtered by title and abstract and then reviewed in full for inclusion and exclusion criteria. Inclusion criteria required that (1) studies compared outcomes for RSSH and RMPH, (2) hysterectomy was indicated for endometrial cancer or hyperplasia with atypia, and (3) studies were available in English. Excluded studies (1) compared single-site and multiport laparoscopic approaches, (2) compared robotic approaches to laparoscopic or abdominal (open) techniques, and (3) employed hysterectomy for benign conditions. Publication bias was assessed using the Egger Regression Correlation analysis. Four studies complied with the selection criteria, comprising 138 patients in the RSSH group and 259 in the RMPH group. Similar outcomes were noted across all measures, including conversion rate (relative risk [RR] = 1.84 and confidence interval [CI] = 0.99-3.43), blood loss (Cohen’s *d* = 1.05 and *Z* = 18.62), operating time (Cohen’s *d *= 0.29 and *Z *= 4.38), and length of hospital stay (Cohen’s *d *= 1.06 and *Z *= 3.86). Publication bias was deemed minimal as indicated by Egger regression values of less than 0.05. These findings suggest that either a surgical approach or AEH with the proper standard of care can provide patients with endometrial cancer.

## Introduction and background

Endometrial cancer is the most common gynecologic malignancy in the United States, with a lifetime occurrence risk of approximately 2.8% [1]. While the pathogenesis of endometrial cancer is not fully understood, advanced age, genetic predisposition (e.g., Lynch syndrome), and unopposed estrogen exposure have all been implicated in its development [1]. Additionally, endometrial cancer can arise from precancerous lesions known as intraepithelial endometrial neoplasia or atypical endometrial hyperplasia (AEH). These neoplasms are believed to carry a 30% to 50% risk of transforming into endometrial cancer [1,2].

Traditionally, endometrial cancer has been classified in concordance with the International Federation of Gynecology and Obstetrics (FIGO) guidelines, which separate endometrial cancer into four stages [3]. Stage I tumors are confined to uterine tissue, with stage IA tumors invading less than half of the myometrium and stage IB tumors invading half the myometrium or greater. Stage II tumors involve the cervical stroma without an extension beyond the uterus itself. Finally, stages III and IV describe worsening tumor invasion to surrounding tissues and/or lymph nodes [3,4]. Such classification aims to provide guidance on endometrial cancer treatment and prognosis [3-5].

When detected early, AEH and endometrial cancer can often be managed surgically. Total hysterectomy with bilateral salpingo-oophorectomy (total abdominal hysterectomy [TAH] with bilateral salpingo-oophorectomy [BSO]) is considered the gold standard for surgical staging of endometrial carcinoma. In low-grade neoplasia, this procedure is also usually curative [5,6]. Minimally invasive surgery is the preferred surgical approach to hysterectomy among experts in gynecology and oncology [5,7]. This surgical approach, including laparoscopic and robotic modalities, has been shown to yield shorter hospital stays, fewer perioperative complications, and better cosmetic results than laparotomy [1]. When comparing robotic and laparoscopic surgical modalities, numerous advantages and disadvantages have been identified. In a study spanning various surgical specialties, laparoscopic procedures have been associated with reduced operating costs [8]. Similar studies have found improved total operating time and complication rates with a laparoscopic approach [9,10]. Research favoring a robotic approach has noted a decreased length of stay with this intervention [9]. Robotic surgery has also been shown to offer improved visibility of the surgical field and greater surgical precision [11]. Despite these proposed advantages and disadvantages, surgical outcomes between robotic and laparoscopic approaches are comparable. A meta-analysis comparing robotic and laparoscopic approaches to hysterectomy for benign conditions found no significant differences in length of stay, estimated blood loss, and intraoperative and postoperative complications [12,13]. Additionally, a retrospective cohort study of 1,150 women found no difference in progression-free survival or overall survival in women undergoing surgery for endometrial cancer via robotic-assisted or traditional laparoscopy [14].

Recent advances in technology have facilitated the performance of both laparoscopic and robotic single-site surgery, in which only one abdominal port is placed to accommodate multiple tools. The robotic single-site modality has been proven a feasible approach to hysterectomy [15-18]. When compared to a traditional multiport approach, robotic single-site surgery has been shown to have reduced hospital costs and increased cosmetic satisfaction in the setting of hysterectomy for benign conditions [19-21]. However, it has also been associated with reduced surgical dexterity and longer operating times [22,23]. Specialists have endorsed robotic single-site hysterectomy (RSSH) for the treatment of gynecologic malignancies, but it remains unclear if RSSH improves surgical outcomes in comparison to traditional robotic multiport hysterectomy (RMPH) in the setting of AEH and endometrial cancer [24,25]. This systematic review and meta-analysis aims to summarize current literature comparing RSSH and RMPH for the treatment of AEH and endometrial cancer to guide clinical practices and promote patients' well-being.

## Review

Methods

Literature Search 

The Preferred Reporting Items for Systematic Reviews and Meta-Analyses (PRISMA) 2020 Checklist guided our literature review [26]. Two authors (ES and NL) conducted their search from June 2022 to October 2022. First, MEDLINE was accessed via PubMed. This search entailed combinations of medical subject headings (MeSH), including "Uterine Neoplasms,” “Endometrial Neoplasms,” “Robotic Surgical Procedures,” and “Treatment Outcome.” Next, a search was conducted utilizing combinations of keywords. We searched (endometrial cancer) AND (hysterectomy), (endometrial hyperplasia) AND (hysterectomy), (endometrial malignancy) AND (hysterectomy), (endometrial cancer) AND (robotic hysterectomy), (endometrial hyperplasia) AND (robotic hysterectomy), and (endometrial malignancy) AND (robotic hysterectomy). Cochrane Library was then accessed via Wiley using these same combinations. Finally, Clinicaltrials.gov was then searched. “Endometrial cancer” was entered as the “condition or disease” and “robotic hysterectomy” as an “additional search term.” A second search utilized “endometrial hyperplasia” as the condition and “robotic hysterectomy” as an “additional search term.” Across all databases, searches were not limited by date of publication or study type (e.g., case reports and randomized controlled trials).

*Inclusion and Exclusion Criteria* 

The original search resulted in 59 articles. Duplicates were removed, and articles were then screened by title and abstract by authors ES and NL independently. Articles were then filtered by inclusion and exclusion criteria. Inclusion criteria required that (1) studies compared outcomes for RSSH and RMPH, (2) hysterectomy was indicated for endometrial cancer or hyperplasia with atypia, and (3) studies were available in English. Included studies were not limited by the preoperative stage or grade of endometrial cancer. Excluded studies (1) compared single-site and multiport laparoscopic approaches, (2) compared robotic approaches to laparoscopic or abdominal (open) techniques, or (3) employed hysterectomy for benign conditions. 

Data Extraction 

**Figure 1:**
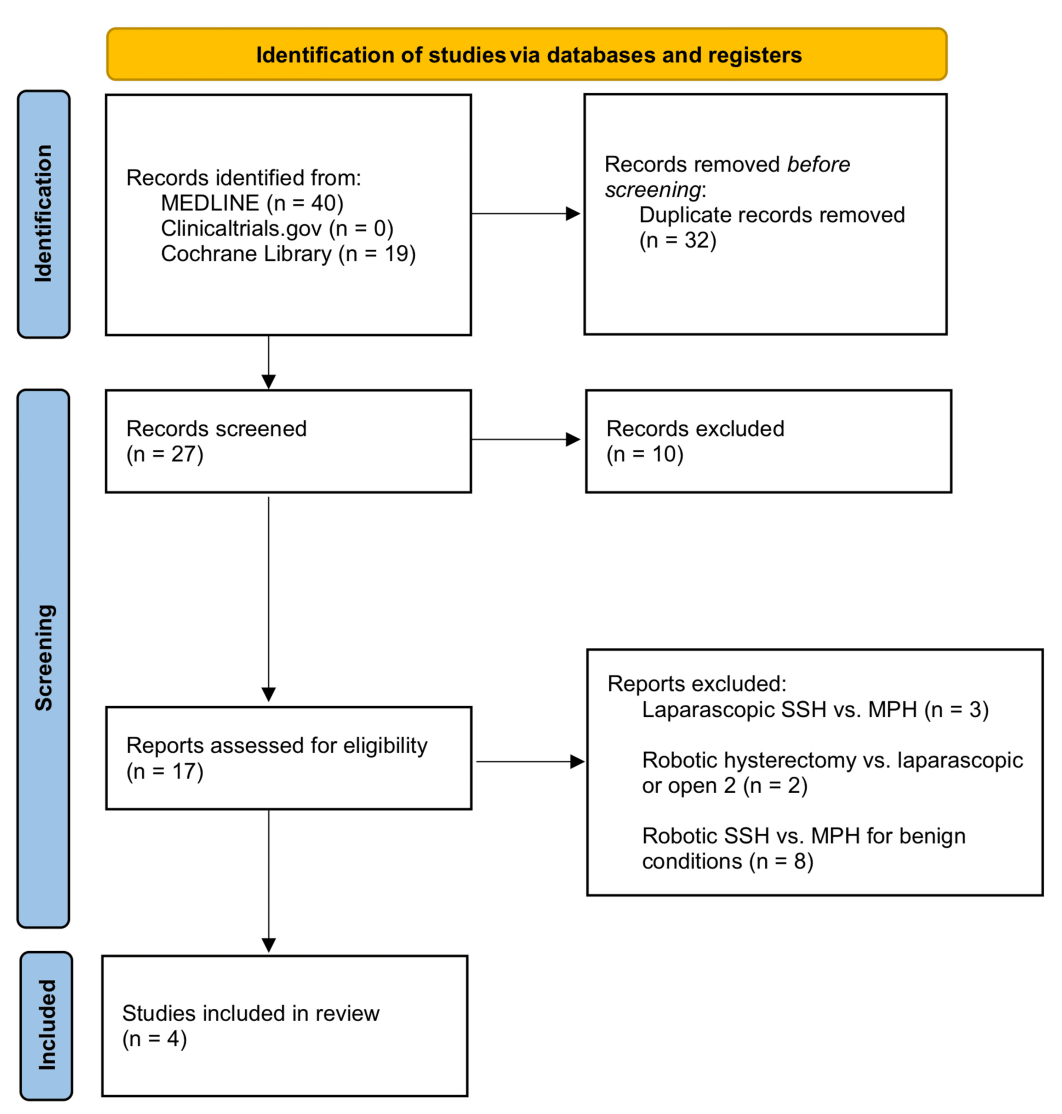
PRISMA 2020 flowchart Source: [26]. SSH, single-site hysterectomy; MPH, multiport hysterectomy; PRISMA, Preferred Reporting Items for Systematic Reviews and Meta-Analyses

Three reviewers (ES, NL, and JC) analyzed articles that met the inclusion criteria for quality. Quality measures included (1) incorporation of a clear population-intervention-control-outcome (PICO) statement, (2) proper interpretation of data (as based on *P*-values and confidence intervals [CIs]), and (3) inclusion of key information. The PICO statement, which guided our review, defined the *population* as patients with biopsy-confirmed endometrial cancer or AEH (regardless of stage or grade). *RSSH* comprised the intervention group and *RMPH* the control group. Outcomes included conversion rate, blood loss, operating time, length of stay, and complications (intraoperative and postoperative). Key information for our review included authors, year of publication, number of participants in experimental and control groups, and experimental findings. Relevant data was entered into a shared document for analysis. A summary of this process can be found in Figure 1. 

*Meta-Analysis* 

Calculations were made using Meta-Essentials Microsoft Excel Workbooks [27]. For qualitative outcomes (i.e., conversion rate), Workbook 2: *Differences Between Independent Groups - Binary Data* was used and relative risks (RRs) and CI were evaluated. An RR value greater than 1.0 and a CI not inclusive of 1.0 were considered significant. For quantitative outcomes (i.e., blood loss), Workbook 3: *Differences Between Independent Groups - Continuous Data* was used and Cohen’s *d* and *Z*-scores were analyzed. Reported medians and interquartile ranges were first converted to mean and standard deviation values using Microsoft Excel to facilitate meta-analysis computations. A value of Cohen’s *d* of greater than 1.0 was considered significant. Larger *Z*-scores were considered reflective of larger differences between groups. Publication bias was quantitatively assessed via the Egger regression model, with a *P*-value of 0.05. All calculations followed a random effects model with 95% CI.

Results

Included Studies 

Four studies met the criteria and were included in our meta-analysis. These studies comprised 138 patients in the RSSH group and 259 in the RMPH group. Studies by Corrado et al. [28], Corrado et al. [29], and Mereu et al. [30] followed a case-control format, whereas the study by Moukarzel et al. [31] utilized a retrospective cohort format. Two of the studies involved concomitant lymph node removal with hysterectomies [28,29]. Publication bias was considered minimal across studies, as Egger regression correlational values across all evaluated outcomes were greater than 0.05.

Included studies varied in patient population and intervention. Corrado et al. compared outcomes for patients undergoing RSSH and RMPH for treatment only stage I endometrial cancer (International Federation of Gynecology and Obstetrics [FIGO] stage IA or IB) with surgical intervention via a singular surgeon at a singular institution [28]. Corrado et al. included patients with stage I and II endometrial cancer, spanning multiple institutions [29]. Mereu et al. evaluated outcomes for patients undergoing robotic hysterectomy for AEH (42.1%), stage I (IA or IB) endometrial cancer (55.3%), and other stages (2.6%). The *other stages* were not specified in their publication. Surgical intervention was performed by four surgeons over multiple institutions [30]. Finally, Moukarzel et al. analyzed outcomes for patients with AEH (26%) or stage I endometrial cancer (IA or IB) (74%) with surgical intervention by three surgeons at a single institution [31]. All the studies found nearly identical surgical outcomes between RSSH and RMPH and could not strongly argue for the utilization of one technique over another. Study characteristics are listed in Table 1. Findings regarding specific outcomes are detailed later.

**Table 1:** Summary of included literature. FIGO, International Federation of Gynecology and Obstetrics

Author (Year)	Country	Study type	Patient population	+/- Lymph node removal	Notes
Corrado et al. (2016) [28]	Italy	Retrospective case-control	FIGO stage IA or IB	Both	Same surgeon and same institution
Corrado et al. (2020) [29]	Italy	Retrospective case-control	FIGO stages I to II	Both	Multiple surgeons and institutions
Mereu et al. (2019) [30]	Italy	Prospective case-control	Atypical endometrial hyperplasia (42.1%), FIGO stage IA or IB (55.3%), and other stages (2.6%)	Yes	Multiple institutions and four surgeons
Moukarzel et al. (2017) [31]	United States	Retrospective cohort	Atypical endometrial hyperplasia (26%) and FIGO stage IA or IB (74%)	Yes	Single institution and three surgeons

Conversion Rate 

Conversion rate, the intraoperative transition from a robotic procedure to an alternative surgical approach, was reported by three studies [28,29,31]. Two studies reported that no patients required a conversion in both single-port and multiport approaches [28,31]. Corrado et al. noted an insignificant difference in conversion between single-port and multiport approaches (*P *= 0.31) [29]. Meta-analysis calculations revealed a positive correlation between conversion rate and RMPH (RR = 1.84); however, the significance of this conclusion is limited, as indicated by a CI of 0.99 to 3.43. Data is shown in Table 2.

**Table 2:** Conversion rate data.

Author (Year)	Single-port patients (n1)	Single port, converted (a)	Single port, not converted (b)	Multiport patients (n2)	Multiport, converted (c)	Multiport, not converted (d)	Significant difference (Yes/No)
Corrado et al. (2016) [28]	23	0	23	46	0	46	No
Corrado et al. (2020) [29]	76	5	71	149	5	144	No, *P *= 0.31
Moukarzel et al. (2017) [31]	14	0	14	13	0	13	No

*Blood Loss* 

Quantitative blood loss was reported by three studies [28,29,31]. A significant decrease in blood loss was noted with RSSH compared to RMPH by Corrado et al. [28,29]. Moukarzel et al. found no significant difference between the two surgical approaches with an estimated blood loss of 50 mL for both [31]. When combined for meta-analysis, similar blood loss was noted between groups (Cohen’s *d *= 1.05 and *Z *= 18.62). Data is shown in Table 3.

**Table 3:** Blood loss (in milliliters) data. RSSH, robotic single-site hysterectomy

Author (Year)	Single port, mean (SD)	Multiport, mean (SD)	Single port (n1)	Multiport (n2)	Significant (Yes/No)
Corrado et al. (2016) [28]	65.65 (35)	115.32 (60)	23	46	Yes, *P *= 0.001, RSSH favored
Corrado et al. (2020) [29]	90 (40)	165 (81.67)	76	149	Yes, *P *= 0.001, RSSH favored
Moukarzel et al. (2017) [31]	52.68 (22.5)	160.38 (122.5)	14	13	No, *P *= 1.0

*Operating Time* 

All four studies reported shorter operating times in the RSSH groups; however, this value was not significant in any of the studies. Meta-analysis calculations revealed Cohen’s *d *of 0.29 and a *Z*-value of 4.38, reflecting an insignificant difference in operating times. Data is shown in Table 4.

**Table 4:** Operating time data (minutes).

Author (Year)	Single port, mean (SD)	Multiport, mean (SD)	Single port (n1)	Multiport (n2)	Significant (Yes/No)
Corrado et al. (2016) [28]	110 (25)	112.7 (26.25)	23	46	No, *P *= 0.889
Corrado et al. (2020) [29]	160.75 (33.67)	178.75 (52.5)	76	149	No, *P *= 0.39
Mereu et al. (2019) [30]	148.7 (18.7)	158.2 (47.6)	25	51	No, *P *= 0.247
Moukarzel et al. (2017) [31]	183.04 (20)	187.23 (36)	14	13	No, *P *= 0.61

Length of Hospital Stay 

Three studies reported a length of a hospital stay beyond 24 hours, and all found significantly shorter stays in the RSSH group [28-30]. However, when synthesized through meta-analysis calculations, nearly equivalent hospital stays were noted (Cohen’s *d* = 1.06 and *Z*-value = 3.86). Data is shown in Table 5.

**Table 5:** Length-of-stay data (days). RSSH, robotic single-site hysterectomy

Author (Year)	Single port, mean (SD)	Multiport, mean (SD)	Single port (n1)	Multiport (n2)	Significant (Yes/No)
Corrado et al. (2016) [28]	2.78 (0.75)	3.51 (1)	23	46	Yes, *P *= 0.001, RSSH favored
Corrado et al. (2020) [29]	2.25 (0.5)	4 (1.33)	76	149	Yes, *P *= 0.007, RSSH favored
Mereu et al. (2019) [30]	2.1 (0.6)	3.1 (1.6)	25	51	Yes, *P* < 0.001, RSSH favored

Complications 

Intraoperative and postoperative complications varied among studies and between surgical approaches. The most common intraoperative complication was vaginal trauma, closely followed by incidental cystotomy and bowel trauma. Postoperative fever occurred in only four patients. Of note, three umbilical hernias were discovered in the RMPH approach, compared to zero found in the RSSH group [29].

Discussion

It has been proposed that RSSH offers numerous benefits over a multiport approach, including reduced costs and greater cosmetic satisfaction [19-21]. While RSSH has been utilized successfully in the treatment of malignant conditions, it has been unclear if RSSH is superior to RMPH in surgical outcomes [24,25]. Through systematic review and meta-analysis, we found that RSSH and RMPH yield similar results. Regarding conversion rate, a significant RR was noted (1.84); however, the CI spanning 1.0 made this value inconclusive. Similarly, when comparing blood loss and length of hospital stay, values of Cohen’s *d* were significant (1.05 and 1.06, respectively), but with a margin too slim to argue in favor of one approach. These values generally corroborate the findings of the individual included studies, none of which found one operative technique significantly superior.

Our research also explored the proposed benefits of RSSH, namely, reduced cost and improved cosmetic satisfaction; however, not enough data was provided by included studies to argue for statistical significance [19-21]. Only the study by Mereu et al. evaluated cosmetic outcomes, finding no differences between the RSSH and RMPH groups [30]. Similarly, only two studies performed cost analysis. Moukarzel et al. noted a significantly reduced cost in the RSSH group (*P *= 0.05) [31]. Corrado et al. also found reduced cost in this group (US$ 5,181.06 vs. US$ 7,772.15); however, a *P*-value was not provided to indicate significance [28].

There were numerous disadvantages to our systematic review and meta-analysis, primarily, the lack of available studies. While research comparing RSSH and RMPH has been prominent in the setting of benign gynecologic disease, the analysis for endometrial cancer and AEH has been much more limited. Such restriction yielded small sample sizes for meta-analysis. The review could have been expanded to gray literature (presentations, posters, abstracts, etc.); however, we questioned the quality of such works, as they often have not undergone peer review or formal evaluation.

Within available data, there has been debate over the role of lymph node resection in the staging and treatment of endometrial cancer and AEH. Although once considered a useful intervention for evaluating disease progression, studies have shown that early detected endometrial cancer infrequently involves the lymph nodes. Additionally, the technical difficulty and risk of postoperative lymphatic congestion controvert routine lymph node resection. Such debate has resulted in a variation of clinical practices across institutions [7]. Because of such conflicts, differences in lymph node removal for RSSH and RMPH were not compared in our analysis.

Furthermore, the format of the presented data posed another hurdle to our research. Across all studies, quantitative data was provided in the format of median values with interquartile ranges. To facilitate meta-analysis, these values were converted to means and standard deviations via Microsoft Excel, which may have contributed to potential skew to our data. It is also important to acknowledge the role of patients' BMI in data calculations. The study by Corrado et al. included only patients with BMI > 30.0 for both the RSSH and RMPH interventions (group A = 30-34.9; group B = 35-39.9; and group C = >40) [29]. Yet, BMI varied within and between other studies: Corrado et al. [28] with RSSH = 26.6 versus RMPH 28.5; Mereu et al. [30] with RSSH = 24.8 versus RMPH 29; and Moukarzel et al. with RSSH= 24.4 vs. RMPH= 27.2 [31]. Thus, it is unclear whether this variable contributed to bias in the meta-analysis calculations.

Overall, despite limitations, we believe our systematic review and meta-analysis to be reflective of current literature comparing surgical outcomes for RSSH and RMPH. Our findings suggest that conversion rate, blood loss, operative times, and length of hospital stay are comparable between these surgical techniques. These findings suggest that either surgical approach can provide patients with endometrial cancer or AEH with the proper standard of care. Additionally, our work highlights the need for further research to determine the optimal surgical protocol for the treatment of endometrial cancer and AEH.

## Conclusions

RSSH offers a novel approach to the surgical management of a gynecologic disease. While research comparing RSSH and RMPH is limited, our systematic review and meta-analysis reveals similar conversion rates, blood loss, operating times, and length of hospital stay between these two groups. We believe further research is indicated to establish the optimal hysterectomy approach for the treatment of endometrial cancer and AEH. 
