# Correction: Surgical Outcomes Following Robotic Single-Site Versus Multiport Hysterectomy for Treatment of Endometrial Cancer: A Systematic Review and Meta-Analysis

**DOI:** 10.7759/cureus.c256

**Published:** 2025-08-26

**Authors:** Emma Schnittka, Nick W Lanpher, Jessica Cushing-murray, Trevor Decker, Praful G Patel

**Affiliations:** 1 Medicine, Alabama College of Osteopathic Medicine, Dothan, USA; 2 Obstetrics and Gynecology, Southeast Health Medical Center, Dothan, USA; 3 Obstetrics and Gynecology, Alabama College of Osteopathic Medicine, Dothan, USA

This article has been corrected to fix the following typo in the PRISMA diagram:

"Duplicates removed before screening" has been corrected from 22 to 32.

